# Virtual Embodiment Using 180° Stereoscopic Video

**DOI:** 10.3389/fpsyg.2020.01229

**Published:** 2020-07-07

**Authors:** Daniel H. Landau, Béatrice S. Hasler, Doron Friedman

**Affiliations:** ^1^Sammy Ofer School of Communications, Interdisciplinary Center Herzliya, Herzliya, Israel; ^2^Department of Media, School of Arts, Design and Architecture, Aalto University, Espoo, Finland

**Keywords:** virtual reality, virtual embodiment, body ownership illusion, 180° video, self-transformation, cognition, time perception

## Abstract

One of the most exciting possibilities of virtual reality is inducing in participants the illusion of owning a virtual body. This has become an established methodological paradigm allowing the study of the psychological and neural correlates of various scenarios that are impossible in the real world, such as gender or age switching. Thus far, full-body ownership illusions have been implemented by using real-time body tracking and avatars based on computer-generated imagery (CGI). We propose an alternative technique to induce perceived ownership over a (photorealistic) virtual body using 180° stereoscopic video, synchronous touch, and narration. We describe the technical components of our novel technique and an example implementation as part of a science–art project that enables participants to experience virtual bodies of different ages, and present the results of an experimental evaluation study based on this experience. Consistent with previous virtual embodiment studies using CGI-based techniques, we found that participants accept a photorealistic virtual body as their own irrespective of its appearance as indicated by similar ratings of the strength of body ownership over a virtual body of a child versus an adult. We further show that our novel technique can alter participants’ cognition in accordance with the characteristics of their virtual body. Specifically, young adult participants who were embodied in the virtual body of a child significantly overestimated the duration of the virtual reality experience compared to a control group who was embodied in a virtual body of their own age. This finding corresponds to chronological age differences in time estimations and extends previous research on virtual child embodiment. Overall, these findings provide initial evidence for the potential of our novel technique to create photorealistic embodiment experiences with comparable psychological effects as have been found using CGI-based techniques while reducing the costs and technical complexity in the production and application of virtual body ownership illusions.

## Introduction

The rubber hand illusion (Botvinick and Cohen, 1998) introduced the possibility of inducing the illusion of perceiving a synthetic arm as one’s own. This illusion has been generalized to a full-body ownership illusion using virtual reality (VR) (Slater et al., 2009). This technique is based on computer-generated imagery (CGI), which substitutes the participant’s physical body with a virtual body. It is achieved by wearing a VR headset and a motion capture suit, and through the real-time mapping of the participant’s body movements onto a virtual character (avatar). This virtual body substitutes the participants’ physical body, and is seen from a first-person perspective (1PP) when they look down toward themselves or view the reflection of their virtual body in a virtual mirror. The co-location of the physical and virtual body and visuomotor synchrony between the movements of the participant’s physical body and the virtual body elicit the illusion of owning the virtual body (Gonzalez-Franco et al., 2010; Kokkinara and Slater, 2014). This body ownership illusion has been shown to be further enhanced through synchronous touch on the participants’ physical body that is visualized on the virtual body (i.e., visuotactile stimulation) (Slater et al., 2010; Maselli and Slater, 2013).

Virtual body ownership has become an established methodological paradigm in neuroscience and psychology, as it allows one to systematically explore the neural, behavioral, and psychological mechanisms underlying body ownership (Ehrsson, 2007; Blanke and Metzinger, 2009). Furthermore, research has shown that changing people’s bodily representations in VR may lead to a profound impact on their self-perception, attitudes, and behaviors (for a review, see Slater and Sanchez-Vives, 2014). For example, Banakou et al. (2013) found that adult participants who embodied a computer-generated virtual body of a child associated more child-like attributes to themselves as measured using an implicit association test and overestimated the sizes of objects compared to participants who were embodied in an adult virtual body. Moreover, such virtual self-transformations have been found to result in more positive evaluations of the social or racial group represented by the virtual body and have been promoted as an effective method to combat prejudice and stereotypes (for reviews, see Maister et al., 2015; Farmer and Maister, 2017). An essential factor that enables such effects is that participants are willing to accept a virtual body as their own irrespective of how similar it looks to their own physical body; previous research has provided evidence that CGI-based embodiment techniques are capable of inducing a sense of body ownership irrespective of the characteristics of the virtual body. For example, studies have shown that light-skinned participants are equally willing to accept a light-skinned or dark-skinned (e.g., Hasler et al., 2017), or even a purple virtual body as their own (Peck et al., 2013). Likewise, no significant differences were found in Bakakou et al.’s (2013) study regarding the strength of the perceived body ownership illusion over a virtual child or adult body.

Although commercial hardware and software components required to create CGI-based body ownership illusions have become available in recent years, it is still not a standard “off the shelf” solution, and requires technical knowhow to produce (Spanlang et al., 2014). This limits a widespread application of the virtual body ownership paradigm, which is currently only used in few research laboratories around the world. Moreover, despite recent advancements in computer graphics toward more human-like virtual characters, the virtual bodies commonly used for creating full-body ownership illusions in research settings do not reach a high degree of resemblance to real humans (yet), which limits the potential for inducing a photorealistic body transfer. While current CGI methods allow for rendering photorealistic animated avatars, these are typically not easy to produce and require special expertise, motion capture hardware and software. Moreover, due to the requirements of fixed high frame rate in immersive VR, photorealism is still very difficult to achieve in VR. It is noteworthy that such powerful psychological effects have been found using CGI-based embodiment techniques despite the lack of photorealism. It is possible that the effects may be even stronger when the virtual bodies become indistinguishable from real human bodies. Alternatively, it could be that a highly photorealistic body would reduce or eliminate the ownership illusion, similar to the “uncanny valley” effect (Mori, 1970; Seyama and Nagayama, 2007).

Alternative techniques have been developed using regular, two-dimensional video, such as the “enfacement” illusion (Porciello et al., 2018) to experimentally induce a photorealistic illusion of owning another person’s face, or techniques used to simulate out-of-body experiences (Ehrsson, 2007). The “enfacement” illusion paradigm uses video recording of an actor who is looking at the camera while his or her face is being stroked. An experimenter performs the same stroke movements synchronously on the participant’s face who views the recorded video on a computer screen in an attempt to simulate a mirror. Out-of-body experiences have been created using video cameras placed behind the participant’s back and this video feed is played in real time, stereoscopically, in a VR headset, such that participants have the illusion of standing behind themselves. Although these video-based paradigms offer a photorealistic representation of a virtual body or face, they do not create a *first-person* embodiment experience in which participants see a virtual body replacing their physical body when looking down toward themselves. These paradigms thus allow for experimentation in illusions of bodily location (Ehrsson, 2007) or self-projection (Porciello et al., 2018) but not bodily identity. We are aware of only one previous attempt to create photorealistic first-person full-body ownership experiences by the artistic productions of the BeAnotherLab.^1^ Their technique relies on switching camera views using VR headsets and requires the presence of two individuals whose bodies are being swapped and who are instructed to synchronize their body movements in real time. As this technique is limited to live performances with synchronization, it is not suitable to deliver standardized embodiment experiences, and to our knowledge, it has not been studied scientifically yet.

We propose an alternative approach for creating photorealistic body ownership illusions in VR using 180° video techniques, synchronous touch, and context-specific narration. We suggest that immersive video (such as 360° or, in our case, 180°) should be regarded as a specific subset of immersive VR as it includes sensorimotor contingencies (Slater, 2009)—one of the most important principles of VR; that is, the visual field changes in accordance with the participant’s head movements. Hence, immersive video is a legitimate technique to produce and study VR experiences, and it is by default photorealistic. It requires a different production pipeline that is often lower in cost and easier. Importantly, it has opened VR to a new community of filmmakers and artists, and allows VR production without any background in programming, 3D modeling, or 3D animation. Due to the popularity of immersive video techniques, we suggest that it is of interest to study if and how this technique can be used to produce first-person embodiment experiences. Many immersive videos nowadays are produced with the intention to induce empathy with specific individuals or social groups. Oftentimes, these immersive video experiences are virtual encounters with the target individual or a representative of the target group (e.g., Schutte and Stilinović, 2017). Few of these experiences are filmed from a first-person view of the target (e.g., Weinel et al., 2018), but we are not aware of any attempts in synchronizing the virtual body of the target with the participant’s body in order to induce a sense of body ownership. Introducing first-person body ownership in such immersive video experiences may have a more powerful impact on the participant.

The main drawback of immersive video is that it is very limited in terms of its interaction capabilities, and participants are not able to navigate or freely explore the virtual environment. Since immersive video experiences are pre-recorded, our approach is only applicable to pre-defined scenarios. This limitation applies not only to the type of scenarios that are possible using video-based techniques but also to the ability of freely moving a pre-recorded virtual body, and visuomotor synchrony has been identified as a critical component to elicit full body ownership illusions (Gonzalez-Franco et al., 2010; Kokkinara and Slater, 2014). However, other embodiment techniques, such as the rubber hand illusion and the enfacement illusion, do not include visuomotor synchrony (i.e., participants cannot move their real hand or head, respectively, during the embodiment experience) and have nevertheless shown to induce a sense of ownership over the artificial body part.

Our main contribution is in introducing this new embodiment technique with all required details and validating it in an experimental study. The main goals of the validation study are to examine (1) the extent to which participants are willing to accept a photorealistic virtual body as their own irrespective of its appearance (as an important pre-condition for embodiment research), and (2) whether similar psychological effects can be obtained that have been commonly found using CGI-based embodiment techniques despite the differences between our immersive video-based approach and the CGI-based approaches. Additionally, we present the opportunity of partially circumventing the limitations of the proposed technique by adding a context-specific narrative layer, which can be used to manipulate the participant into performing specific motions in specific moments as a means to introduce limited visuomotor synchrony.

Section “Materials and Equipment” provides a description of the technical components of the proposed 180° video embodiment technique, and in section “First Implementation of the 180° Stereoscopic Video Embodiment Technique: The ‘Time-Body’ Artistic Experience,” we present its first implementation in a science–art project—a progressive virtual aging experience—from childhood to adulthood and old age. In section “Evaluation Study,” we present the method and results of the validation study of the proposed video embodiment technique regarding its potential to generate the expected psychological effects. We conclude with a discussion (section “Discussion”) of the methodological validity and practical implications of our 180° video-based technique compared to the CGI-based technique that is commonly used to create first-person full-body ownership illusions.

## Materials and Equipment

### Components of the 180° Stereoscopic Video-Based Virtual Embodiment

The proposed 180° video-based embodiment technique consists of three main components: (1) first-person view of the virtual body, (2) synchronous visuo-tactile stimulation, and (3) a narrative layer with context-specific “passive” motor actions.

#### First-Person View of the Virtual Body

**FIGURE 1:**
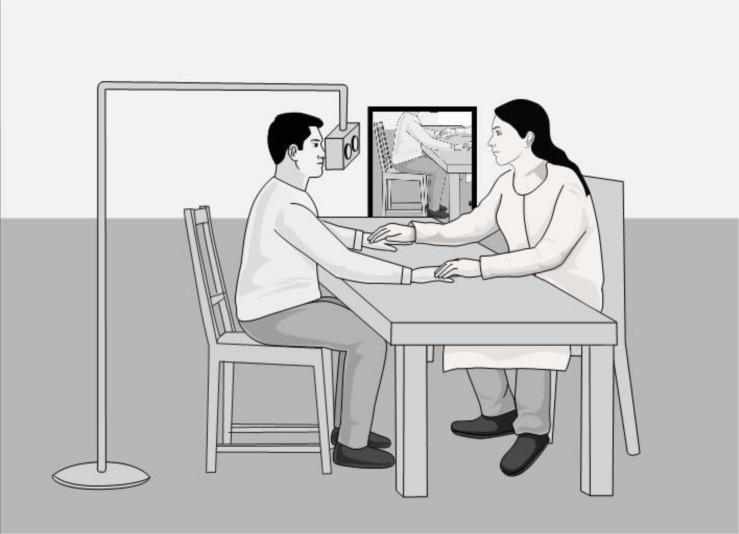
The technical setup of the 1PP shoot.

In order to produce a first-person view of the virtual body, the target person (a human actor) is filmed in 1PP who later serves as the virtual body of the participant. The target person is filmed by placing a stereoscopic 180° camera rig with 220° fisheye lenses in front of the target person’s eyes (see Figure 1).

The target person is seated at a table with his or her hands placed on it. A mirror can be placed to the left or right of the target person so that the camera captures the reflection of the body without showing the face. Not filming the target person’s face serves two goals: (1) it reduces the perceived difference in appearance between the participant and the target person, which may facilitate self-identification with the surrogate body, and (2) it makes it possible for participants to rotate their heads freely without “disconnecting” from the virtual body (e.g., avoiding such cases as when the virtual body is looking forward while the participant is turning the head sideways).

#### Synchronous Visuotactile Stimulation

**FIGURE 2:**
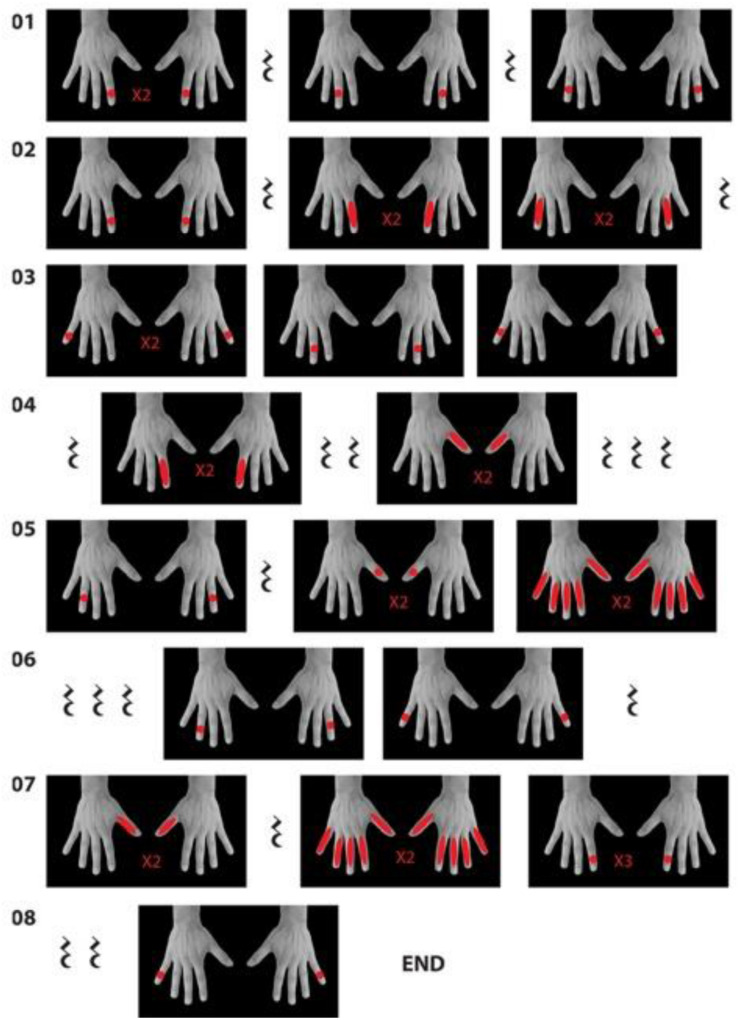
The score for the touch sequence.

The second major component of the 180° video-based embodiment technique is synchronous human touch, which is an integral component in related techniques, such as the rubber hand illusion (Botvinick and Cohen, 1998), the “enfacement” illusion (Porciello et al., 2018), as well as video-based techniques used to elicit out-of-body experiences (Ehrsson, 2007), and is often applied in CGI-based full-body ownership studies as well (e.g., Maselli and Slater, 2013). Another person who serves as a virtual interlocutor with the participant in the embodiment experience is seated in front of the target person on the other side of the table, and performs a 30-s sequence of tapping and stroking movements on the hands of the target person—equivalent to the induction of the rubber hand illusion. The purpose of this procedure is to establish a visuo-tactile sensorimotor contingency (Slater et al., 2010) whereby the participant not only sees another person’s body replacing their own, but also sees the touch on that person’s hands and feels synchronous touch on their own hands. The tapping sequence is performed to the sound of an 80-beat-per-minute (BPM) metronome to ensure accurately timed tapping and stroking. The sequence of tapping and stroking instructions may be transcribed similarly to a musical score (see Figure 2). This score later serves a human performer during the embodiment experience as an instruction for the touch sequence to indicate the timing, which finger to touch, and the type of touch (tap or stroke) to perform. In the example illustrated in Figure 2, the red dots indicate tapping and the red lines indicate a long stroke. The musical sign of a rest indicates a pause in the touch sequence. In addition to performing the touch sequence, the interlocutor may speak to the target person and provide verbal instructions (later experienced as personal instructions by the participant in the embodiment session) to look down at their hands and to their left to see the virtual body in the mirror.

#### Narrative Layer and Context-Specific “Passive” Motor Actions

Following the touch sequence, which aims to establish the illusion of virtual body ownership, we propose to add a narrative layer as a third component. The narrative context may vary depending on the type of body and the intended purpose of the embodiment experience. Enriching this experience beyond a purely perceptual illusion on the bodily level through a narrative context may increase engagement as has been shown in studies on VR-based storytelling (Shin, 2018), and possibly create a more profound and long-lasting impact on the participant. As part of the narrative layer, motor actions can be performed, such as placing context-specific objects into the target person’s hands or guiding their hands or arms into certain positions or performing certain motions and gestures, such as a handshake. These pre-recorded interactions between the virtual interlocutor and the target person are later performed during the embodiment experience by the experimenter on the participant’s arms or hands synchronously to the motor actions of the virtual interlocutor and the target person in the video. Such passive forms of inducing visuomotor correlations partially compensate for the lack of agency (i.e., lack of control over the virtual body and inability to freely explore the virtual environment). Hence, the narrative layer fulfills two purposes: (1) adding contextual meaning to the embodiment experience through storytelling, and (2) partially circumventing technical limitations of the video-based embodiment technique.

### Embodiment Experience

To embody participants in the body of the target person, the 180° stereoscopic video is played back using a VR headset and headphones. The participants are seated at a table and place their hands in the same location on the table where the virtual hands appear. They are instructed not to initiate hand movements but are encouraged to turn their heads to look around. The experimenter sits on the opposite side of the table where the virtual interlocutor in the video appears, and hears the sound of the video simultaneously in his headphones. The experimenter performs the tap and stroke movements that the participant sees in the video in complete synchronization with the 80 BPM metronome beat following the performance scores illustrated in Figure 2. It is critical to apply the visuotactile stimulation in perfect synchronization as even slight deviations (i.e., asynchronous strokes) can break the body ownership illusion, which is a well-studied factor in CGI-based embodiment studies (see Kokkinara and Slater, 2014). If a narrative layer is added to the experience that requires additional interactions with the participant, such as placing objects in participant’s hands, the experimenter also carries out these actions in synchrony with the virtual interlocutor whom the participant sees in front of them in the video. As for any other type of visuo-tactile stimulation (i.e., synchronous touch and stroke movements) these passive motor actions must be performed in complete synchronization.

## First Implementation of the 180° Stereoscopic Video Embodiment Technique: the “Time-Body” Artistic Experience

**FIGURE 3:**
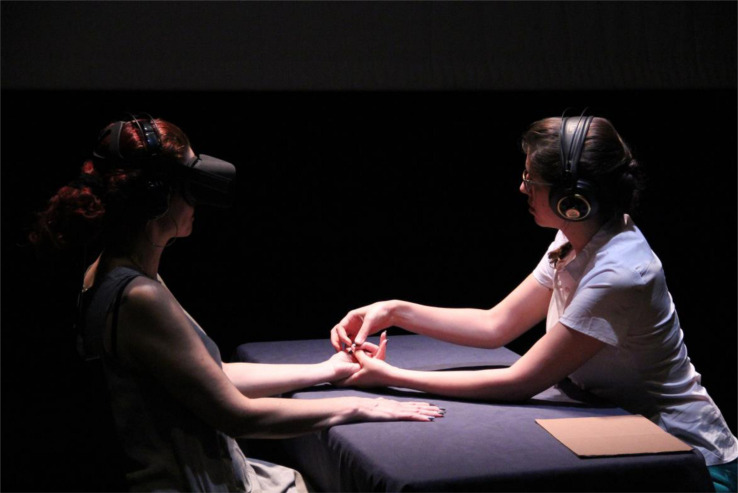
Time-Body performed at an art festival. Photo by Ben Hertzog.

The first implementation of the 180° video embodiment technique was a performative experiment called Time-Body Study^2^ by the first author (DHL); it was performed in the PrintScreen Festival for Digital-Culture (Israel), in June 2016 (Figure 3). The goal of this artistic experience was to create an embodied narrative experience of the progression of time over a person’s lifespan. It provides the participant with the experience of growing older through a stage-wise embodiment in the virtual body of a 7-year-old, a 40-year-old, and an 80-year-old. Two target persons (one male and one female) were filmed as representatives of the three ages (i.e., child, adult, and elderly) in order to allow for a gender-congruent embodiment experience. In the following, we describe the Time-Body experience from the perspective of the participant, which was generated according to the procedure described in section “Materials and Equipment.”

In a first step, we filmed the target person in 1PP who later serves as the virtual body of the participant. The target person was filmed by placing a stereoscopic 180° camera rig (manufacturer: ZCam E1) with 220° fisheye lenses (manufacturer: iZugar) in front of the target person’s eyes (see Figure 1). The footage was edited using Adobe Premier and the color was adjusted using Adobe After Effects.

**FIGURE 4:**
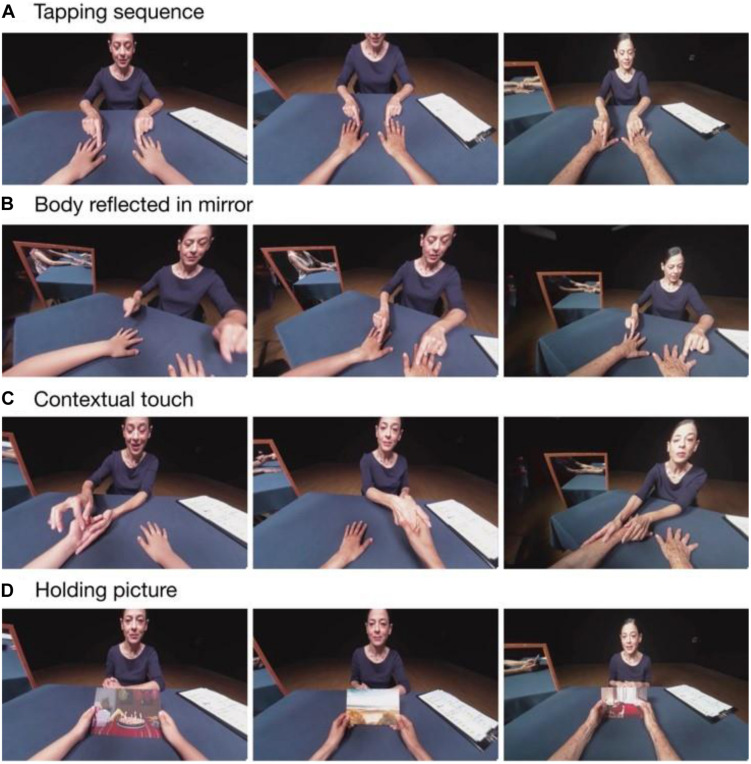
Screenshots of the Time-Body experience. The rows contain screenshots from each of the three ages (child, adult, and elderly) from left to right. **(A)** Filmed actress tapping the sequence on the 1PP hands. **(B)** The mirror placed on the left reveals the re-embodied body of the participant. **(C)** The actress performs touch sequences in the context of each age. **(D)** The actress places a picture in the hands of the 1PP person. The actress gave permission for the publication of this image.

A woman (virtual interlocutor) on the other side of the table addressed the participant with “You are {7/40/80} years old” and prompted them to look at their hands. Then, she performed the initial 30-s tapping and stroking sequence (Figure 4A) on the hands of the filmed target person, which is carried out simultaneously by the performer on the participant’s hands. During the pauses, the interlocutor asks the participant to look at the mirror to their left, where they could see their “new” body (Figure 4B). After the touch sequence, the interlocutor describes a brief episode of the embodied person’s life and performs a context-specific touch gesture (Figure 4C): (1) the “this little piggy went to market” game for the child’s body, (2) a business handshake for the adult’s body, and (3) a comforting touch for the elderly person’s body. These actions are synchronously carried out by the performer. At each age, the interlocutor places a photograph in the hands of the filmed target person. The physical object is blank, but in the video, the photographs are related to the age-specific narrative, and the interlocutor tells an age-specific story relating to the image in the photo (Figure 4D): (1) birthday party for the 7-year-old, (2) a walk on the beach with a friend for the 40-year-old, and (3) an intimate moment with a close family member after returning from a cemetery for the elderly person. We constructed the story in such a way that it is possible to play this narrative experience forward or backward either as one storyline (i.e., age progression or regression) or separately using only one of the three ages.

## Evaluation Study

The evaluation study addresses two essential aspects of virtual body ownership illusions: the ability to (1) create a strong sense of ownership over a virtual body irrespective of its appearance, and (2) affect participants’ cognition in accordance with the characteristics of the virtual body. Although photorealism is expected to make embodiment experiences feel more realistic, it is possible that participants are less willing to accept a photorealistic body as their own as it actually represents the body of another human individual. Embodiment of a computer-generated virtual character, on the other hand, may lead to greater suspension of disbelief. The more a photorealistic body differs from the appearance of the participant’s own physical body, for example, regarding its age, the more difficult it may be to accept it as one’s own.

Such potential differences in how participants perceive—and accept—a photorealistic virtual body as their own would also influence the extent to which similar effects on participants’ cognition and behavior can be generated with our proposed technique as have been found in previous studies using CGI-based embodiment techniques. The current study addressed this issue by aiming to replicate and extend previous findings on the cognitive impact of embodying a virtual body of a different age. Particularly, we investigated whether our technique can lead to similar effects as have been found in a previous virtual child embodiment study by Banakou et al. (2013). While they examined how embodying adult participants in a computer-generated virtual child body affects their spatial cognition, we tested the effect on temporal cognition in the current study. Specifically, we examined whether adult participants who had been embodied in the photorealistic body of the 7-year-old using the 180° video-based embodiment technique as part of the Time-Body experience would overestimate the duration of the VR experience compared to a control group who had been embodied in a virtual body of their own age. We used retrospective time estimation (i.e., judgment of past periods of time) as the measure of interest as it is commonly assumed that time is perceived as passing faster as we grow older. Indeed, research has shown that children tend to overestimate the duration of a past temporal stimulus or event compared to adults (e.g., McCormack et al., 1999; Droit-Volet et al., 2007; Droit-Volet, 2013). These age differences in estimating time of temporal stimuli or time intervals have been attributed to developmental changes in the allocation of attention to duration during such tasks (Droit-Volet et al., 2006). While children have more difficulties focusing their attention to durations of tasks, this ability has been found to improve with chronological age. Importantly, in Banakou et al.’s (2013) study, the overestimation of object sizes in the child embodiment condition was not due to simply having a shorter virtual body. Participants who were embodied in a short adult body (of the size of a child) did not overestimate the sizes of objects. Hence, the effect was likely due to the attribution of child-like attributes to the self in the child embodiment condition. Based on this previous finding, we hypothesize that the same effects may be obtained regarding time estimation (i.e., longer estimation of the duration of the VR experience as compared to a control group) when embodying adult participants in the (photorealistic) virtual body of a child compared to embodying the virtual body of an adult.

### Participants

Eighty-three students (38 men, 45 women) between the age of 19 and 31 years (*M* = 23.55, SD = 2.67) at the Interdisciplinary Center (IDC) Herzliya, Israel, participated in the evaluation study. Participants were randomly assigned to one of two conditions: Embodiment in the photorealistic virtual body of a 7-year-old child (v7) (*n* = 33) or of a 25-year old (v25) (*n* = 50) (control group).^3^ Both conditions were taken from the original Time-Body experience (see section “First Implementation of the 180° Stereoscopic Video Embodiment Technique: The ‘Time-Body’ Artistic Experience”). Since participants were in their early to mid-20s, we created a corresponding control condition by having the virtual interlocutor in the video refer to the participant as a 25-year-old instead of a 40-year-old but using the same virtual body of the 40-year old model as in the original Time-Body experience. This led to a believable embodiment experience of a 25-year old since only the upper body and arms were visible but not the face of the target person. In both conditions, the virtual body matched participants’ gender. All participants volunteered to take part in the experiment and received credits for their participation. The study was approved by the ethics committee of IDC Herzliya.

### Procedure

After signing an informed consent form, participants completed a short demographic questionnaire. Then they were seated at a table and were instructed to place their hands on it, positioned exactly where the target person’s hands in the Time-Body experience were placed. The experimenter instructed participants to actively look around during the VR experience but not to initiate hand movements. He then helped the participants put on the VR headset. The 180° video of the respective age condition was then played back while the experimenter performed all the touch sequences in complete synchronization. The VR experience in both conditions lasted exactly 3 min. Subsequently, participants completed a questionnaire, which first asked them to estimate the duration of the VR experience, followed by measures of body ownership.

### Measurements

#### Time Estimation

Participants were asked to estimate the duration of the VR experience in minutes and seconds from the moment they put on the VR headset until the time when they took it off. Time estimations above 180 s are an overestimation of the actual duration of the experience, while time estimations below the actual duration are considered as underestimation.

#### Body Ownership

In order to measure the strength of perceived body ownership, we adapted the body ownership questionnaire employed by Banakou et al. (2013) as well as other previous CGI-based full-body ownership studies (e.g., Peck et al., 2013; Hasler et al., 2017). It consists of three items: (1) *To what extent did you feel that the body you saw when you looked down at yourself was your own body?* (VRBody), (2) *To what extent did you feel that the body you saw when you looked at yourself in the mirror was your own body?* (Mirror), and (3) *To what extent did you feel as if you had two bodies?* (TwoBodies) (reverse-coded). These are the items that are most commonly used in previous embodiment studies (see Gonzalez-Franco and Peck, 2018): The VRBody item has been used in 100% of the previous studies; and the TwoBodies item in 66% of previous studies. The Mirror item is commonly used in embodiment studies that use a virtual mirror. Participants viewed their virtual body in a seated position with their hands placed on a table, and mainly saw the arms of their virtual body when they looked down at themselves. Therefore, we adjusted the wording of the first item to: *To what extent did you feel that the hands you saw when you looked down at yourself were your own hands?* The other items were used in their original wording. All items were rated on a scale from 1 (not at all) to 5 (very much).

### Results

#### Body Ownership

**FIGURE 5:**
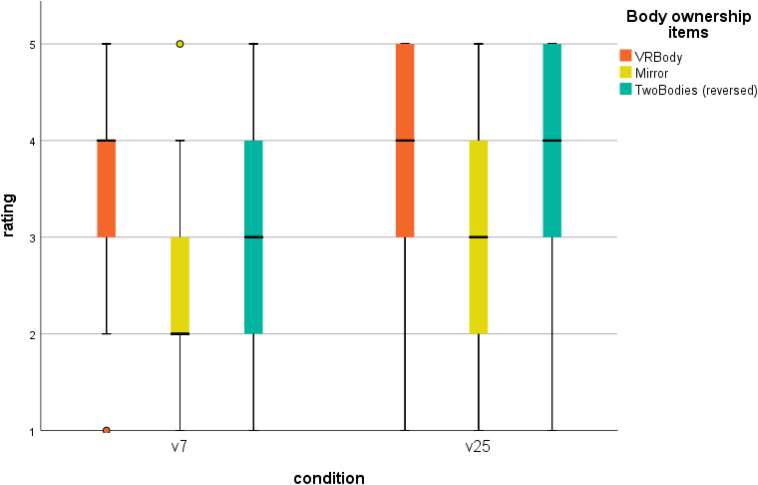
Boxplot of body ownership ratings by condition and items.

The distribution of the body ownership ratings on each of the three items for the v7 and v25 conditions is shown in Figure 5. Mann–Whitney *U* tests revealed no statistically significant differences between the v7 and the v25 conditions on any of the body ownership items; VRBody: *U* = 950.5, *SE* = 103.12, *p* = 0.22, Mirror: *U* = 908, *SE* = 104.35, *p* = 0.43, TwoBodies (reversed): *U* = 977, *SE* = 104.35, *p* = 0.15. Hence, our interpretation is that participants accepted the virtual body as their own irrespective of whether the body represented a child or an adult. We note the lower ratings on the Mirror item compared to the other two items. Related-samples Wilcoxon signed rank tests indicate significant median differences between the Mirror item and both the VRBody item, *Z* = 5.39, *SE* = 156.47, *p* < 0.001, and the reversed TwoBodies item, *Z* = 3.61, *SE* = 136.89, *p* < 0.001.

#### Time Estimation

**FIGURE 6:**
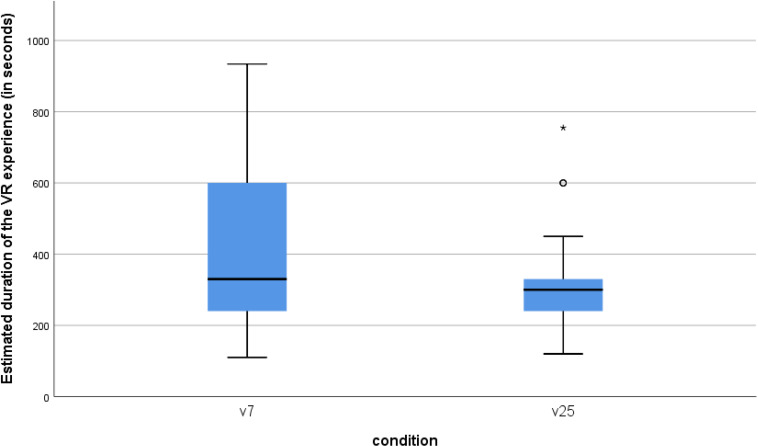
Boxplot of the estimated duration of the VR experience (in seconds) for the virtual child (v7) and the virtual adult (v25) conditions. The actual duration was 180 s.

In both conditions, participants tended to overestimate the duration of the VR experience, which lasted exactly 180 s in both conditions (see Figure 6). However, as hypothesized, those who were embodied in the body of the 7-year-old (v7) estimated the duration of the VR experience as significantly longer (*M* = 411.88 s, *SD* = 211.61 s) than participants in the control group (v25) (*M* = 312.35 s, *SD* = 122.91 s), *t*(80) = 2.69, *p* = 0.009, CI = [25.93, 173.14], *d* = 0.58; *F*(1,79) = 7.15, *p* = 0.009 when controlling for participants’ actual age. After removing an extreme outlier in the v25 condition with a time estimate of 755 s, the analysis revealed an even stronger effect: *t*(79) = 3.06, *p* = 0.003, CI = [37.89, 179.62], *d* = 0.65; *F*(1,78) = 9.23, *p* = 0.003, when controlling for age.

## Discussion

The current paper introduces a novel technique to induce a photorealistic body ownership illusion using 180° stereoscopic video in combination with synchronous touch and a narrative layer. We describe a first example illustrating how this technique can be applied to create the illusion of owning a photorealistic virtual body of a child, adult, or an elderly. The results of our evaluation study provide initial evidence that this new technique fulfills two major criteria of the virtual body ownership illusion paradigm that are essential for its application as a valid research method in the fields of psychology and neuroscience: (1) it induces a sense of body ownership irrespective of how similar the virtual body is to the participant’s physical body, and (2) it is capable of creating a significant impact on participants’ cognition in accordance with the characteristics of the virtual body. We first discuss each of these core findings in more detail and provide suggestions for future research. Then, we turn to a broader discussion of the contribution of our new technique to the advancement of embodiment research and alternative types of applications.

### Body Ownership: Comparison of Body Ownership Ratings Depending on the Virtual Body’s Appearance

As hypothesized, we found no statistically significant differences regarding the strength of perceived body ownership over the virtual body of a child versus an adult virtual body using our new 180° video-based embodiment technique. This finding is crucial for the application of our new technique in research on the plasticity of the self that requires participants’ willingness to accept a virtual body as their own irrespective of its appearance. Moreover, the evaluation study showed that the strength of perceived body ownership over photorealistic virtual bodies is around the same levels as typically reported in virtual embodiment studies using CGI-based techniques (e.g., Banakou et al., 2013; Peck et al., 2013; Hasler et al., 2017). However, we found significantly lower ratings on a body ownership item (Mirror) that refers to the extent to which participants perceived the virtual body they saw in the mirror as their own. This may be due to the technical limitation of video-based embodiment that does not allow for agency; that is, the participant cannot control the virtual body since its movements are pre-recorded. People are used to performing movements in front of a mirror in their everyday lives. It is possible that seeing their virtual body in a mirror and remembering that they are instructed not to initiate any movements other than moving their head made participants aware that they do not have control over the virtual body.

While immersive video is inherently linear and strictly limits the possibilities for interaction, we show how visuotactile synchrony with a virtual body can be achieved using synchronous touch and predefined motions as part of the narrative layer. Such passive motor actions (i.e., guiding the participants’ arms or hands into certain positions or gestures in synchrony with the pre-recorded movements of the virtual body) can partially overcome the lack of agency in the proposed technique. This procedure allows for simple interactions without breaking the body ownership illusion. The participants, without exceptions, adjusted their hand motions and positions to those of their virtual body, whenever such motions occurred. Thus, the physical and virtual hands remained aligned during these interactions. However, participants are in a passive role during these interactions as they do not self-initiate these hand movements but are guided by the virtual interlocutor or experimenter, respectively. It is possible that these limitations are reflected in the lower ratings on the Mirror item.

There are other ways to create an illusion of agency, for example, by letting participants play a mirror game (Noy et al., 2011) in which they are requested to synchronize their hand movements with pre-recorded (slow) movements of their virtual hands. Such alternative techniques to induce a sense of agency provide a greater degree of freedom (i.e., participants are less passive) but also bear the risk of leading to deviations from the pre-recorded movements of the virtual body, which is likely to break the body ownership illusion. Nevertheless, it is worth exploring such alternative motor action techniques in future implementations of the 180° video-based embodiment technique.

### Psychological Effects of Altered Body Representation: The Impact of Virtual Child Embodiment on Time Estimation

Besides creating the perceptual illusion of owning another body, participants are expected to change their cognition in accordance with the characteristics of the virtual body. The findings of our evaluation study indicate that 180° video-based embodiment can generate such psychological effects. Specifically, young adult participants who were embodied in a virtual body of a 7-year-old child estimated the VR experience as lasting significantly longer compared to those who were embodied in a virtual body of their own age. This finding is consistent with age differences in time estimation (Droit-Volet et al., 2006), and complements previous work on virtual child embodiment by Banakou et al. (2013) who found similar effects using CGI-based embodiment techniques. While this previous study showed an impact on size estimations of objects in the child embodiment condition, our finding shows that this phenomenon extends to time estimations. However, it remains unclear whether our findings were due to actual changes in participants’ attention to task durations in the child embodiment condition (i.e., the effect was due to the assumed underlying mechanism of developmental changes in time estimations), or alternatively, whether participants merely acted according to their lay theory about childhood (i.e., that time is passing slower as a child). Further research is required in order to examine the underlying mechanism behind the impact of virtual child embodiment on time estimation, and such research may also consider examining this effect using alternative methods, such as the common paradigms of temporal bisection and temporal generalization tasks (see Droit-Volet et al., 2006).

Moreover, since the virtual embodiment experiences differed not only regarding the appearance of the virtual body (child vs. adult), but also regarding the content of the narration and contextual gestures that were applied, it remains unclear what has caused this psychological effect. Based on our current evaluation study, we can only conclude that the overall embodiment experience using the three components (first-person view of the virtual body, synchronous touch, and narrative layer) led to these effects. It is critical for future research to examine each of the three components separately in order to evaluate their respective contribution to the body ownership illusion and the psychological consequences that emerge from it.

### Emotional Impact of Photorealistic Virtual Embodiment

The current evaluation study also did not quantitatively measure another critical and unique factor—the emotional impact of photorealistic embodiment experiences. We observed that the combination of being embodied in a photorealistic virtual body with synchronous touch and narration triggers exceptionally strong emotional reactions, and even led to tears among participants for whom the embodiment experience activated personal (childhood) memories. Similarly, we observed strong emotional responses during the artistic performances of the Time-Body experience that provides a 9-min run-through of the human life cycle from childhood, adulthood, and old age. Although these observations are currently only based on anecdotal evidence, they indicate that this new embodiment technique can potentially create strong emotional experiences with a profound impact on participants. Future research should aim to quantify the emotional impact of this novel type of embodiment experiences, ideally combining self-reports and physiological measures.

### Contribution to Embodiment Research and Artistic Applications

Based on our initial findings, we conclude that 180° video-based embodiment can be a methodologically valid alternative to the common CGI-based embodiment techniques. The next questions we would like to address are the scenarios for which this alternative technique is suitable and how it contributes to the advancement of embodiment research.

While in some cases a first-person body ownership illusion is of interest by itself, in many cases, we want to study how people respond in a social scenario while being embodied in a virtual body that differs from their real, physical appearance in certain characteristics, such as age, race, or gender. The proposed technique naturally allows for designing such (pre-defined) interactions with virtual others. In such cases, there is a major difference between the production of immersive video versus CGI production—photorealistic virtual humans are still difficult and expensive to generate, they would typically require using professional motion capture and software systems, and developing interactive virtual humans is still considered to include several open challenges (e.g., Burden and Savin-Baden, 2019). Moreover, real-time rendering in VR is still challenging due to the need for high fixed frame rate and high resolutions, and photorealistic rendering inside VR is still a challenge. Using immersive video allows for easily incorporating a human actor, as we demonstrated above. The same trade-off as before applies here—immersive video is easier to produce and is by default photorealistic, but interaction is limited.

The ability to create a photorealistic body transfer offers entirely new possibilities, such as the embodiment of particular individuals. This also creates interesting new opportunities for the use of virtual embodiment as a therapeutic tool, for example, in parent–children or couple relationships. As our current study shows, the potential of 180° stereoscopic video-based embodiment is not limited to this particular application. It can also be used as an alternative embodiment technique in research areas that study the psychological and neuroscientific basis of self-representation and self–other relations.

On the practical level, our novel technique has the advantage that it is arguably easier and less costly to produce than an equivalent CGI experience. Once the videos have been generated, they are easy to administer as they do not require participants to wear motion capture suits. Having such an alternative technique at hand that allows for an easy administration of body ownership experiences is an important contribution to the research community as it allows for a more widespread application of the virtual body ownership paradigm in a wide range of research areas. These practical advantages of 180° video-based embodiment also facilitate the application of embodiment experiences in intervention programs outside of the research laboratory, and may also be adopted by other fields, such as education, art, and activism.

## Data Availability Statement

The datasets generated for this study are available on request to the corresponding author.

## Ethics Statement

The studies involving human participants were reviewed and approved by Institutional Review Board of IDC Herzliya. The patients/participants provided their written informed consent to participate in this study.

## Author Contributions

DL and BH equally contributed to the manuscript, designed and conducted the evaluation study. DL conceptualized and implemented the 180° video-based embodiment technique, prepared an initial draft of the manuscript and the artistic Time-Body experience under supervision of DF. BH analyzed the data and wrote the final manuscript. DF provided essential comments on the manuscript. All authors contributed to the article and approved the submitted version.

## Conflict of Interest

The authors declare that the research was conducted in the absence of any commercial or financial relationships that could be construed as a potential conflict of interest.

## Footnotes

1 http://beanotherlab.org/home/work/tmtba/

2 https://vimeo.com/198779184

3 The unequal number of participants in the two conditions is due to an extension of the current study in another research project in which the same control group (v25) was used. Additional measures were collected in the v25 condition for the other project, but they were included in the questionnaire after the time estimation and body ownership measures used in the current study.
